# Oleate alters the immune response in non-small cell lung adenocarcinoma through regulation of HMGB1 release

**DOI:** 10.3389/fcell.2024.1348707

**Published:** 2024-07-19

**Authors:** Breanna Cole-Skinner, Nicole M. Andre, Zachary Blankenheim, Kate M. Root, Kisa Jafri, Glenn E. Simmons

**Affiliations:** ^1^ Department of Molecular Microbiology and Immunology, University of Missouri, Columbia, United States; ^2^ Department of Biomedical Sciences, College of Veterinary Medicine, Cornell University, Ithaca, United States; ^3^ Department of Biomedical Sciences, School of Medicine, University of Minnesota, Duluth, United States

**Keywords:** HMGB1 (high mobility group box 1), lung cancer, tumor microenvironment, fatty acid, immunotherapy

## Abstract

**Background:**

Cancer cell evasion of the immune response is critical to cancer development and metastases. Clinicians' ability to kickstart the immune system to target these rogue cells is an ever-growing area of research and medicine. This study delved into the relationship between lipid metabolism, High Mobility Group Box 1 protein (HMGB1)–a pro-inflammatory damage-associated molecular pattern protein–and immune regulation within non-small cell lung adenocarcinoma (NSCLC).

**Method:**

To address this question, we used a combination of proteomics, molecular biology, and bioinformatic techniques to investigate the relationship between fatty acids and immune signals within NSCLC.

**Results:**

We found that the expression of stearoyl CoA desaturase 1 (SCD1) was decreased in NSCLC tumors compared to normal tissues. This emphasized the critical role of lipid metabolism in tumor progression. Interestingly, monounsaturated fatty acid (MUFA) availability affected the expression of programmed death ligand-1 (PD-L1), a pivotal immune checkpoint target in lung cancer cells and immune cells, as well as HMGB1, suggesting a novel approach to modulating the immune response. This study uncovered a complex interplay between SCD1, PD-L1, and HMGB1, influencing the immunological sensitivity of tumors.

**Conclusion:**

Our work underscores the critical importance of understanding the intricate relationships between lipid metabolism and immune modulation to develop more effective NSCLC treatments and personalized therapies. As we continue to explore these connections, we hope to contribute significantly to the ever-evolving field of cancer research, improving patient outcomes and advancing precision medicine in NSCLC.

## Introduction

Non-small cell lung adenocarcinoma (NSCLC) is still a significant global health concern. As immunotherapeutic treatment options such as immune checkpoint blockade (ICB) become more common, many patients do not benefit from these advancements. As many as 80% of patients recommended for ICB do not respond or become refractory to treatment (Boyero et al., 2020; Tran and Theodorescu, 2020; Wang et al., 2020; Abdayem and Planchard, 2021). Unfortunately, the mechanism behind ICB treatment failure is unknown. This highlights a pressing need for a deeper understanding of the molecular mechanisms governing lung adenocarcinoma progression and its immune microenvironment.

Several studies have underscored the pivotal role of aberrant metabolism in shaping the behavior of cancer cells, with a particular focus on the influence of lipid metabolism (Sun et al., 2015; Peck et al., 2016; Senga et al., 2018; Chen et al., 2019; Zhao et al., 2021). The level of unsaturated fatty acids in the cell alters the expression of many proteins including oncogenes in various cancer models (Kim et al., 2015). In hypermetabolic cancer cells, glycolytic flux increases the production of stearic and palmitic acid due to the excess production of acetyl-CoA. Stearoyl-CoA desaturase (SCD1) the enzyme that synthesize *de novo* monounsaturated fatty acid, converts the accumulating saturated fatty acids to their unsaturated forms before cells succumb to lipotoxicity (Schaffer, 2016). Scientists have been looking to leverage this metabolic bottleneck and view SCD1 as a potential target for anti-cancer therapy. This emerging body of evidence compels us to explore how lipid metabolic alterations impact tumor development and immune responses within the tumor microenvironment of lung cancer.

Earlier studies have shown that High Mobility Group Box 1 protein (HMGB1) drives the activity of the pro-inflammatory transcription factor NF-kB, a pivotal player in the immune response (Chen et al., 2004; Li et al., 2012; Wang et al., 2017). Although HMGB1 participates in the inflammatory response, the release of HMGB1 has also been shown to induce immunosuppression in tumors (Wild et al., 2012; Wang et al., 2019). Interestingly, inhibition of HMGB1 improved therapeutic response (Bonaldi et al., 2003). The secretion of HMGB1 is regulated by post-translational modifications such as phosphorylation, lactylation, and acetylation, which to varying degrees masks the HMGB1 nuclear localization sequence (NLS1 and NLS2), allowing for nuclear-cytoplasmic transport and later release (Bonaldi et al., 2003; Youn and Shin, 2006; Jube et al., 2012; Kim et al., 2016; Yang et al., 2022). Sirtuin deacetylases remove these acetyl groups from HMGB1, preventing release in an unsaturated lipid-dependent manner, illustrating the relationship between HMGB1 and fatty acid metabolism (Kim et al., 2014; Rickenbacher et al., 2014; Hwang et al., 2015; Zhang et al., 2015; Chen et al., 2018). As both fatty acid synthesis and HMGB1 participate in cancer development, we embarked on investigating the mechanistic underpinnings of the relationship between lipid metabolism, immune regulation, and its potential impact on cancer therapy.

In the current study, we examined The Cancer Genome Atlas (TCGA, n = 542) and the Clinical Proteomic Tumor Analysis Consortium (CPTAC, n = 115) Lung adenocarcinoma (LUAD) datasets to view the relationship between lipid metabolism and HMGB1 in NSCLC tumors (Gillette et al., 2020). Previous studies have reported that the presence of unsaturated fatty acids affects the expression of proteins involved in tumor growth (Kim et al., 2015). We focused our analysis on specific proteins in lipogenic and HMGB1/RAGE pathways. Our findings revealed a relationship between SCD1 and HMGB1 protein in the tumors of NSCLC patients. Our *in vitro* studies illustrated that altering SCD1 activity led to changes in HMGB1 release and concomitant changes in the expression of PD-L1 on the surface of lung cancer cells and innate immune cells. Preliminary examination of a few NSCLC patient samples suggested a relationship exists between serum HMGB1 and tumor-associated PD-L1.

Overall, our research unveils an uncharted axis involving MUFA, HMGB1, and PD-L1 in NSCLC by illuminating the intricate lipid metabolism network, immune regulation, and potential therapeutic targets within this challenging disease. These findings show the potential promise of targeting lipid metabolism in precision medicine and developing innovative immunotherapeutic approaches to treat NSCLC.

## Results

### Expression of stearoyl CoA desaturase is decreased in non-small lung adenocarcinoma tumors

**FIGURE 1:**
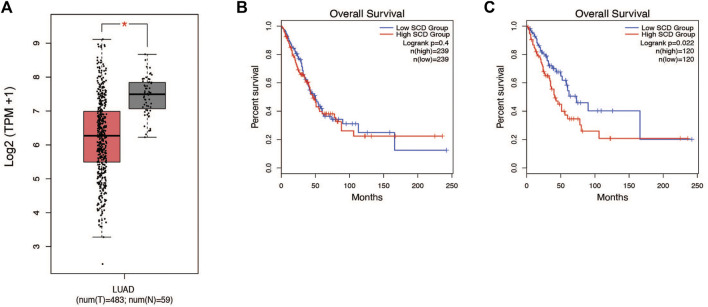
SCD1 gene expression is decreased in NSCLC tumors and associated with survival. **(A)** Analysis of TCGA lung adenocarcinoma RNA-seq datasets from UCSC Xena project comparing SCD1 gene expression in lung tumors vs. normal tissue using GEPIA webserver. **(B)** Kaplan-Meier plot of median overall survival (OS) in groups with either high or low expression of SCD1. Analysis based on gene expression. **(C)** Quartile overall survival in groups with either high or low SCD1 gene expression. For differential gene expression, method for differential analysis is one-way ANOVA, using disease state (Tumor vs. Normal) as variable for calculating differential expression. For Survival plots, a Log-rank test was done to determine significance of the difference in survival between each group.

Patients with lung cancer have been shown to have altered metabolism in critical pathways (Merino Salvador et al., 2017). Earlier studies by our lab and others have shown that alterations in lipid metabolism are often essential to how cancer cells behave (Sun et al., 2015; CD36-Mediated Lipid Metabolism Promotes Metastasis, 2016; Imanikia et al., 2019; Jeffords et al., 2020; Zhao et al., 2021). We were interested in investigating how lipid metabolism is altered within the lung tumors of patients. To address this question, we analyzed the transcriptome from the lung cancer patients (TCGA LUAD, n = 542). Based on our earlier experience we decided to integrate SCD1 into our analysis of the lung cancer cohort (Merino Salvador et al., 2017). Within this patient population, SCD1 gene expression was significantly decreased in lung tumors compared to normal tissue (Figure 1A). Interestingly, the impact of SCD1 gene expression on survival was only clear in the upper and lower quartiles of the population, suggesting an outlier effect may be involved (Figures 1B, C). Still, seeing a significant difference in SCD1 expression between normal and malignant tissues suggests that the activity of SCD1 and *de novo* production of monounsaturated fatty acids (MUFA) takes part in the development of lung tumors.

### Monounsaturated fatty acid promotes differential expression of proteins in lung cancer cells

**FIGURE 2:**
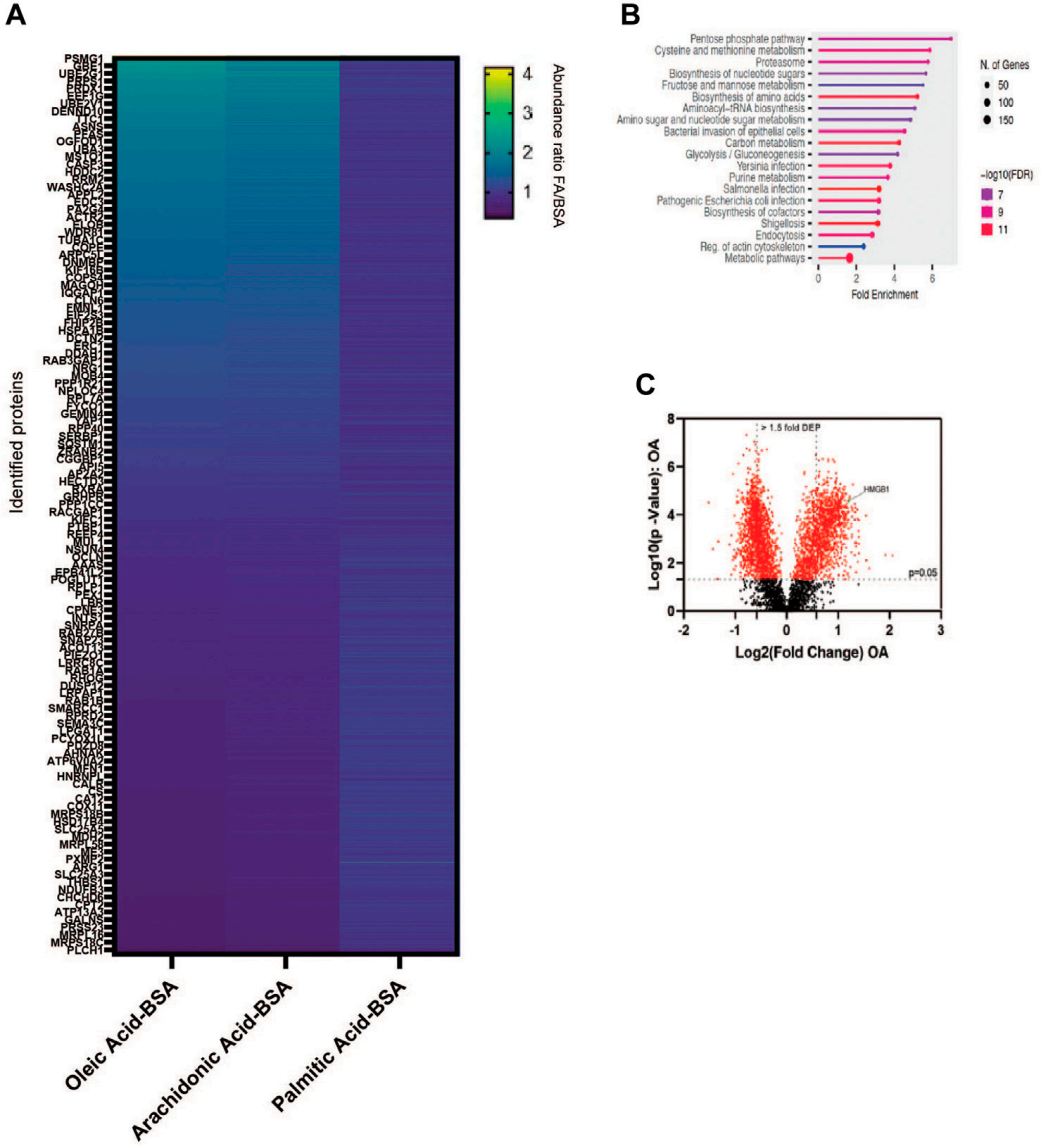
MUFA increases expression of a subset of proteins in NSCLC. **(A)** Overview of proteomic analysis of A549 lung cancer cells following 16-h delipidation and 4-h fatty acid replenishment with either oleate-BSA, arachidonate-BSA, or palmitate-BSA. **(B)** Gene Ontology of unsaturated fatty acid-enriched peptides in A549 Cells using ShinyGO (Ge et al., 2020). **(C)** Volcano plot of differential expressed peptides in A549 lung cancer cells following delipidation and oleate-BSA replenishment.

The production of unsaturated fatty acids is essential for supporting healthy cells. However, during carcinogenesis increases in cellular metabolism, especially glycolysis, increases fatty acid production (Pouyafar et al., 2019). Fatty acid synthesis, by default, produces saturated fat. The accumulation of saturated fat is toxic to cells and requires conversion to an unsaturated fatty acid via stearoyl CoA desaturases (SCD1 and SCD5). Therefore, increased unsaturated fatty acid can result from the increases in glycolysis and lipogenesis. To determine the effect of increased unsaturated fat on lung cancer proteome, we briefly deprived lung adenocarcinoma cells (A549) of exogenous fatty acids using SCD1 inhibitor (A9395762) and delipidated serum, followed by acute exposure to saturated, mono, or poly-unsaturated fatty acids. We quantified the cellular proteome using tandem mass tags, comparing relative expressions across the BSA, Oleate-BSA, Arachidonate-BSA, or Palmitate-BSA treated samples. Approximately 1,000 proteins were enriched in unsaturated (mono and poly) fatty acid-treated samples compared to BSA control (saturated fatty acids had the opposite effect on many of these proteins) (Figure 2A; Table 1). Gene ontology showed that many enriched genes were associated with metabolic pathways and cellular response to infection (Figure 2B). Within the differentially enriched and repressed proteins, one high mobility group box 1 protein (HMGB1) was associated with the immune response and poor prognosis in cancer patients (Figure 2C). Based on this, we investigated the relationship between HMGB1 and lipid metabolism in lung cancer.

**TABLE 1:** List of proteins identified by tandem mass tag (TMT) proteomic analysis of A549 Lung cancer cells treated by oleic acid, arachidonic acid, or palmitic acid following overnight delipidation.

MUFA enriched proteins in A549 lung cells			
Gene symbol	Abundance ratio: (OA)/(BSA)	Abundance ratio: (AA)/(BSA)	Abundance ratio: (PA)/(BSA)
PSMG1	4.157	3.537	1.628
TUBAL3	3.785	2.792	1.757
RNASEH2B	3.042	2.432	1.115
PSMG2	2.919	2.481	1.18
UBE2D3	2.911	2.303	1.072
CASP3	2.644	2.432	1.112
PAPSS1	2.64	2.494	0.977
LASP1	2.637	2.199	1.083
NUCKS1	2.637	2.203	0.885
PEBP1	2.631	2.308	1.156
ERCC1	2.631	1.649	1.098
TAGLN3	2.605	2.126	0.906
SUMO2	2.596	2.075	0.934
DRAP1	2.584	2.467	0.882
PSME2	2.556	2.175	0.984
NACA	2.547	1.897	1.101
TUBB6	2.524	2.17	1.062
UBA2	2.522	2.421	0.775
TKT	2.52	2.185	0.85
TALDO1	2.509	2.432	0.847
ANP32B	2.508	2.375	0.76
ILKAP	2.498	2.832	1.141
PLIN3	2.452	2.549	0.959
EIF5A	2.439	2.085	1.103
HMGB3	2.426	2.092	0.871
STMN1	2.412	2.298	0.969
GNPNAT1	2.411	1.954	1.023
FBXO22	2.409	1.931	1.004
TAGLN2	2.4	2.054	0.991
IAH1	2.394	2.084	1.333
CKS1B	2.392	1.982	1.011
BLVRB	2.386	2.04	1.152
TRAFD1	2.367	2.103	1.218
GGA3	2.339	1.95	0.998
SAE1	2.332	2.136	0.853
SRP19	2.311	1.752	1.191
THOP1	2.31	1.93	1.241
ADSL	2.307	2.006	1.117
HMGB1	2.305	2.124	0.783
PGD	2.298	2.099	1.115
GBE1	2.295	2.09	1.099
FAH	2.288	1.974	1.105
UBE2K	2.284	2.004	1.086
TTC5	2.282	1.784	1.053
ANP32A	2.279	2.228	0.778
PCNA	2.271	1.97	0.958
SNF8	2.269	2.158	1.128
NECAP2	2.268	1.861	1.278
TXNDC9	2.267	1.858	0.991
NANS	2.263	2.191	0.847
CNN2	2.247	2.097	0.757
BOLA2; BOLA2B	2.245	2.044	0.907
DIAPH2	2.235	2.312	0.963
FABP5	2.232	2.137	1.113
EEF2	2.23	2.004	1.065
UBE2N	2.228	2.034	0.921
FAM50A	2.225	2.171	0.831
COMMD7	2.224	1.761	1.046
UBE2Q1	2.223	1.946	1.007
PFN1	2.222	2.053	1.196
RPA3	2.219	1.755	0.919
DCUN1D1	2.218	1.807	0.943
UBE2R2	2.217	2.001	1.081
JPT2	2.215	2.177	0.888
TARS1	2.215	2.034	1.055
MDH1	2.21	2.025	1.147
XPO6	2.209	1.594	0.733
MYL12B	2.204	1.82	0.855
RNF214	2.2	1.656	1.079
UGDH	2.197	1.929	1.001
MYH16	2.197	1.99	0.717
AKR1C2	2.192	1.946	1.03
HPRT1	2.19	1.921	1.151
ALDOA	2.19	1.963	1.073
TBCB	2.189	2.053	1.02
ZFYVE19	2.189	1.517	1.163
UBE2L3	2.188	2.025	1.005
RAN	2.187	1.901	0.955
ZC2HC1A	2.186	2.026	1.115
FBXL18	2.185	1.771	0.998
UBE2G1	2.185	1.917	0.974
TXN	2.184	1.923	1.294
SAR1A	2.182	1.785	1.126
TMA7	2.177	2.335	0.882
MAT2A	2.172	2.136	0.968
SUB1	2.169	2.12	0.905
TIGAR	2.169	2.173	1.016
PTMS	2.168	2.19	0.738
PGAM1	2.165	1.99	1.073
PPP1R14B	2.164	1.973	1.018
GPI	2.16	1.999	1.069
RANBP1	2.16	2.042	0.956
MYH9	2.158	1.861	0.845
TUBB4A	2.157	1.831	1.053
THUMPD1	2.155	1.971	0.795
PSAT1	2.155	2.061	1.004
MAP4K5	2.152	1.851	0.647
PSME3IP1	2.148	2.077	0.815
PCBD1	2.147	2.158	0.912
NARS1	2.144	2.011	0.93
PNP	2.143	1.882	1.059
APRT	2.139	2.093	1.044
PRDX6	2.136	1.993	1.036
LTA4H	2.134	2.022	1.023
YARS1	2.133	2.077	0.987
GSTO1	2.132	2.181	0.99
HSP90AA1	2.128	1.904	1.055
CACYBP	2.126	2.038	0.988
SPATA5L1	2.123	1.776	1.153
CNDP2	2.119	2.043	1.045
TUBB4B	2.118	1.889	1.079
KYNU	2.118	2.059	1.064
ARIH2	2.116	2.194	0.916
SNX6	2.115	1.97	1.018
RGS10	2.114	2.045	0.814
CTPS1	2.111	1.996	1.075
MAPK8	2.11	2.048	1.292
PTGES3	2.102	1.873	1.042
APIP	2.101	1.753	1.066
LPP	2.1	1.838	0.998
PRPS1	2.1	2.026	0.88
PTPA	2.097	1.841	1.085
GDI2	2.095	1.889	1.108

### HMGB1 and SCD1 genes are inversely related in lung cancer patients

**FIGURE 3:**
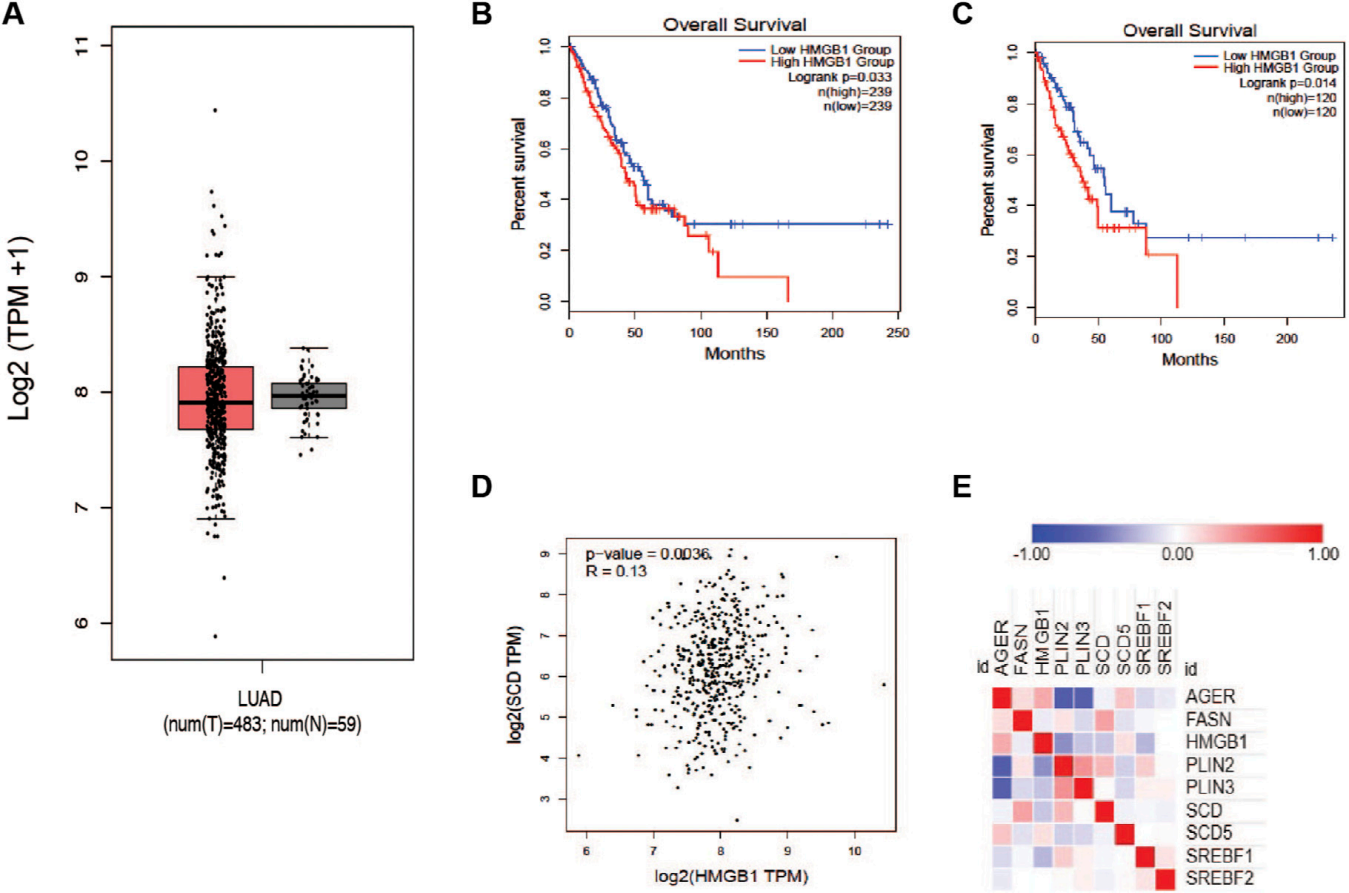
Proteins involved in HMGB1 and MUFA synthetic pathways are inversely correlated in NSCLC. **(A)** Analysis of TCGA lung adenocarcinoma RNA-seq datasets from UCSC Xena project comparing HMGB1 gene expression in lung tumors vs. normal tissue using GEPIA webserver. **(B)** Kaplan-Meier plot of median overall survival (OS) in groups with either high or low expression of HMGB1. **(C)** Quartile overall survival in groups with either high or low HMGB1 gene expression. **(D)** Pearson correlation analysis between the expression of SCD1 and HMGB1 in lung cancer patients. **(E)** Heatmap generated from Clinical Proteomic Tumor Analysis Consortium lung adenocarcinoma dataset comparing the expression proteins in lipogenic (FASN, SREBF1, SREBF2, SCD, SCD5, PLIN2, and PLIN3) and HMGB1 (AGER and HMGB1) pathways.

It has been reported that HMGB1 protein plays a role in cancer progression and response to therapy. Based on earlier reports, we wanted to determine the relationship between HMGB1 and the production of unsaturated fatty acids in tumors. To explore this question, we accessed the TCGA LUAD dataset and plotted the expression of the HMGB1 gene in tumors and normal tissue. There was a trivial difference in the level of HMGB1; however, there was a much larger distribution of expression in lung tumor groups (Figure 3A). Interestingly, patients with elevated HMGB1 had worse survival outcomes compared to the HMGB1 low group (Figures 3B, C). To confirm the presence of a relationship between HMGB1 and SCD1, we measure the correlation between the expression of both transcripts within the LUAD cohort data. A small but significant positive correlation existed between SCD1 and HMGB1 RNA in patients’ tumors (Figure 3D). This seemed counter to our working hypothesis, so we next analyzed the proteome of lung cancer tumors using the CPTAC Apollo dataset. We chose a subset of proteins involved in lipogenesis and lipid packaging, along with HMGB1 and SCD1. HMGB1 protein was inversely related to many lipogenic and lipid packaging proteins, including SCD1 and sterol response element binding factor 1 (SREBF1) (Figure 3E). These results suggest that in tumors, the level of unsaturated fatty acids has a regulatory role in the expression of HMGB1 protein.

### Inhibition of SCD1 promotes the release of HMGB1 protein from cultured lung cancer cells

**FIGURE 4:**
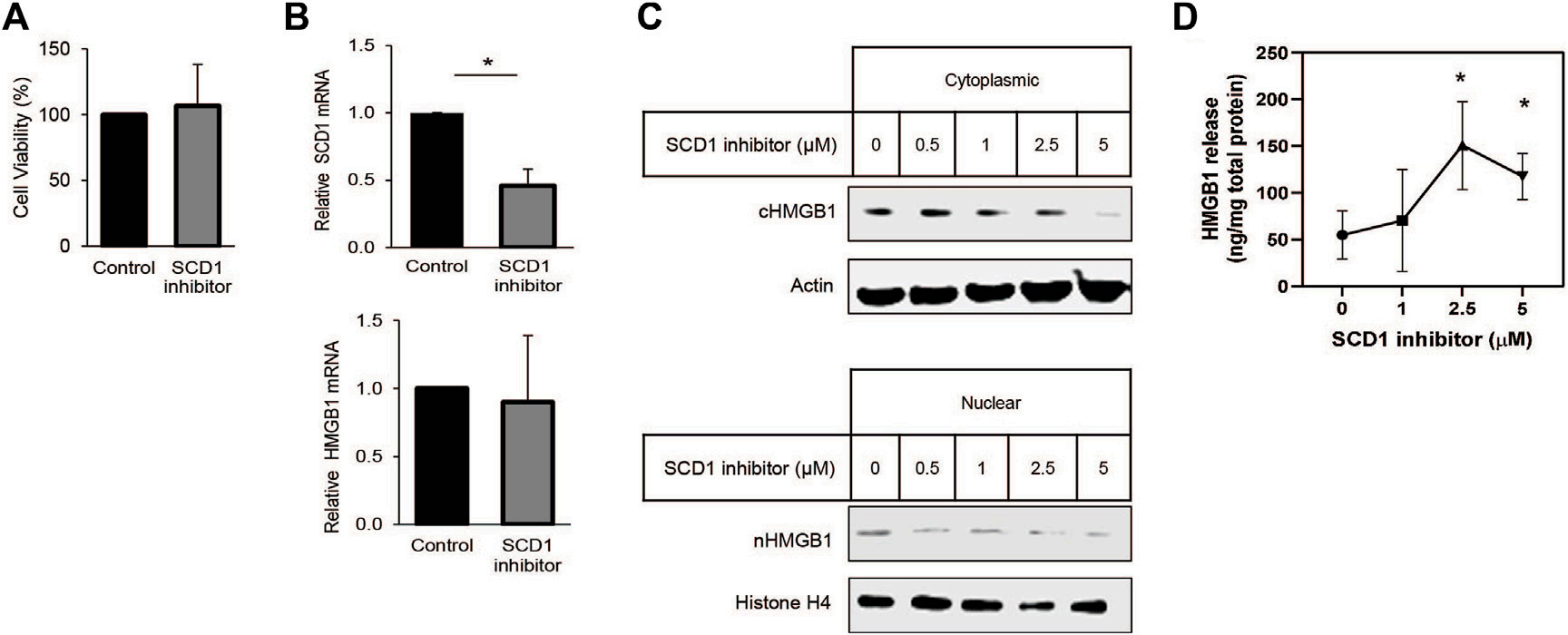
SCD inhibition promotes release of HMGB1 from lung cancer cells. **(A)** Cell-titer Glo viability assay of cells treated for 24 h with 1 µM SCD1 inhibitor, A939572. **(B)** Real-time quantitative PCR analysis for HMGB1 and SCD1 mRNA in A549 cells treated 24 h with 1 µM SCD1 inhibitor. **(C)** Immunoblot analysis of HMGB1 expression in nuclear and cytoplasmic fraction of A549 cells treated 24 h with increasing concentration of SCD1 inhibitor. **(D)** HMGB1-specific enzyme-linked immunosorbent assay on extracellular media from A549 cells treated 24 h with increasing concentration of SCD1 inhibitor. Error bars represent the standard error of the mean (S.E.M.), an * indicates a *p*-value of < 0.05.

Many studies have shown that SCD1 is vital for rapid cell growth. In some instances, SCD1 has been proposed as a target for anti-cancer therapy (Mason et al., 2012; Noto et al., 2013; von Roemeling et al., 2013; Pisanu et al., 2017). Earlier work from our lab has shown that SCD1 expression is correlated with survival in patients with clear cell renal cell carcinoma (ccRCC) (Jeffords et al., 2020). Based on this, we looked to determine how HMGB1 was affected by the inhibition of SCD1. Treating cells with low micromolar amounts of the SCD1 inhibitor did not affect cell viability or HMGB1 RNA levels but significantly decreased SCD1 mRNA (Figures 4A, B). Further, increased concentrations of the SCD1 inhibitor led to a significant dose-dependent decrease in intracellular HMGB1 and a concomitant increase in extracellular HMGB1 (Figures 4C, D). Thus, these data show that SCD1 activity does not affect the transcription of the HMGB1 gene; instead, it affects the localization of HMGB1 within lung cancer cells.

### Monounsaturated fatty acid increases the retention of HMGB1 in lung cancer cells

**FIGURE 5:**
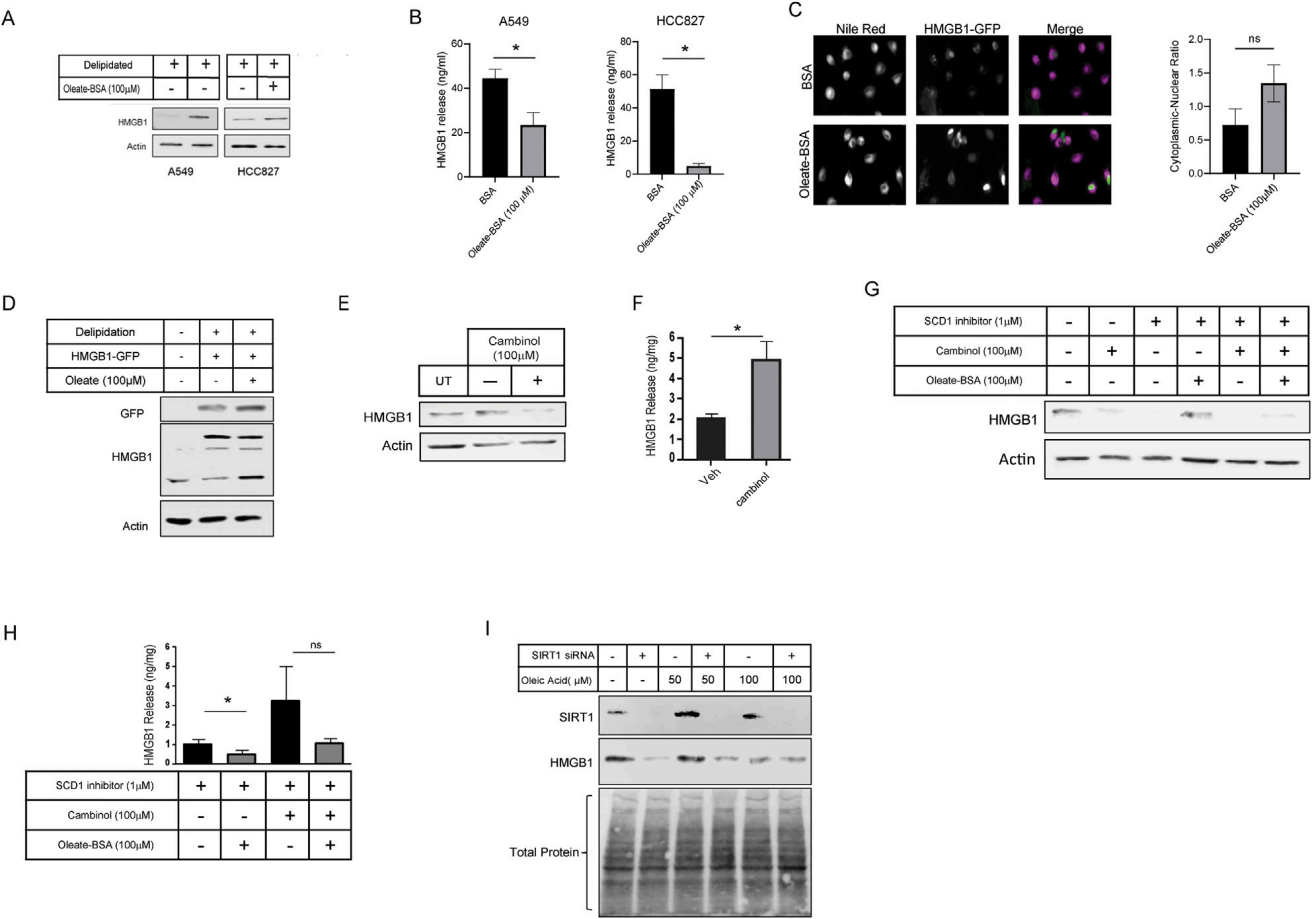
MUFA increases retention of HMGB1 in lung cancer cells in a SIRT-dependent manner. **(A)** Immunoblot protein analysis of KRAS G12S mutant (A549) and EGFR deletion mutant (HCC827) lung cancer cells lines treated with delipidation media (delipidated serum and 1 μM SCD1 inhibitor) and 4-h replenishment with oleate-BSA. **(B)** HMGB1-specific ELISA on extracellular media from A549 and HCC827 lung cancer cells following 16-h delipidation and 4-h oleate-BSA replenishment. **(C)** Fluorescence microscopy of HMGB1-GFP transfected A549 cells following 16-h delipidation and 4-h oleate- BSA replenishment and neutral lipid staining with Nile Red, along with quantitation of localization of HMGB1-GFP signals. **(D)** Immunoblot protein analysis of HMGB1-GFP transfected A549 cells from **(C)**. **(E)** Immunoblot protein analysis of A549 lung cancer cells treated SIRT1 inhibitor cambinol for 24-h. **(F)** HMGB1-specific ELISA on extracellular media from cells treated in **(E)**. **(G)** Immunoblot protein analysis of A549 cells treated with combination of cambinol and SCD1 inhibitor in the presence and oleateBSA. **(H)** HMGB1-specific ELISA on extracellular media obtained from cells treated with SIRT1 and SCD1 inhibitors in **(G)**. **(I)** Immunoblot protein analysis of delipidated A549 cells replenished with oleic acid-BSA, 72 h after transfection with SIRT1-specific siRNA. Error bars represent the standard error of the mean (S.E.M.), an *, indicates a p-value < 0.05.

Since the inhibition of SCD1 appeared to have such an impact on HMGB1 release, we directly investigated the effect of MUFA on HMGB1. Using multiple lung cancer cell lines (A549, HCC827, H1299, and H23), we measured the level of intracellular HMGB1 following our delipidation procedure. When cells were deprived of lipids, HMGB1 release was increased, but the re-introduction of MUFA significantly suppressed HMGB1 release in all cell lines evaluated (Figures 5A, B). To demonstrate how lipid supplementation affected HMGB1 within cells, we transfected GFP-tagged HMGB1 into A549 cells and exposed cells to delipidation and lipid replenishment. From imaging, HMGB1-GFP appeared enriched in the cytoplasm of cells supplemented with MUFA compared to BSA controls (Figure 5C). Although the fluorescent modification increased molecular weight of HMGB1, we confirmed that HMGB1-GFP behaved similarly to what we had observed of endogenous HMGB1 in lung cancer cells (Figure 5D). This suggests that MUFA is either blocking late steps in nuclear-cytoplasm-extracellular secretion or that MUFA is promoting the reuptake of secreted HMGB1.

### MUFA and SIRT1 cooperate to retain HMGB1 inside lung cancer cells

HMGB1 has been proven to move throughout the cell due to post-translational modifications. Several labs have shown acetylation to regulate the ability of HMGB1 to leave the nucleus and exit the cell (Bonaldi et al., 2003; Youn and Shin, 2006; Topalova et al., 2008; Kook et al., 2009). Acetylation of HMGB1 is conducted by a host of acetyltransferases. Conversely, removal of acetyl groups from HMGB1 is conducted by histone deacetylases (HDACs), and in particular one family of HDAC proteins known as sirtuins. Evidence suggests that SIRT1 removes acetyl groups from HMGB1 in a lipid-dependent manner (Hwang et al., 2015; Chen et al., 2018). To determine the role of sirtuin deacetylases in MUFA-dependent regulation of HMGB1 release, we treated A549 cells with sirtuin inhibitor, cambinol, and measured the intracellular as well as the extracellular HMGB1 levels. In the presence of the inhibitor, we noted a decrease in cell associated HMGB1 and a concomitant increase in HMGB1 release (Figures 5E, F). Sirtuin inhibition appeared to stimulate HMGB1 release, but to demonstrate that MUFA was involved, we delipidated cells in the presence and absence of cambinol, followed by brief exposure to MUFA. Following pretreatment with sirtuin inhibitor, the application of MUFA led to a decrease in HMGB1 release and an increase in intracellular protein (Figures 5G, H). To verify the effect of cambinol treatment was due to SIRT1 activity we next knocked down the gene in lung cancer cells. SIRT1 siRNA treatment led to a dramatic decrease in SIRT1 protein, and a concomitant decrease in HMGB1 protein as seen in the cambinol experiments (Figure 5I). We used total protein staining to determine equivalent loading as SIRT1 knockdown noticeably affected actin levels in our hands (not shown). Together, these data are evidence that the relationship between HMGB1 and sirtuin is responsible for the retention of HMGB1 in lung cancer.

### MUFA suppresses NF-kB-dependent cytokine release from lung cancer cells

**FIGURE 6:**
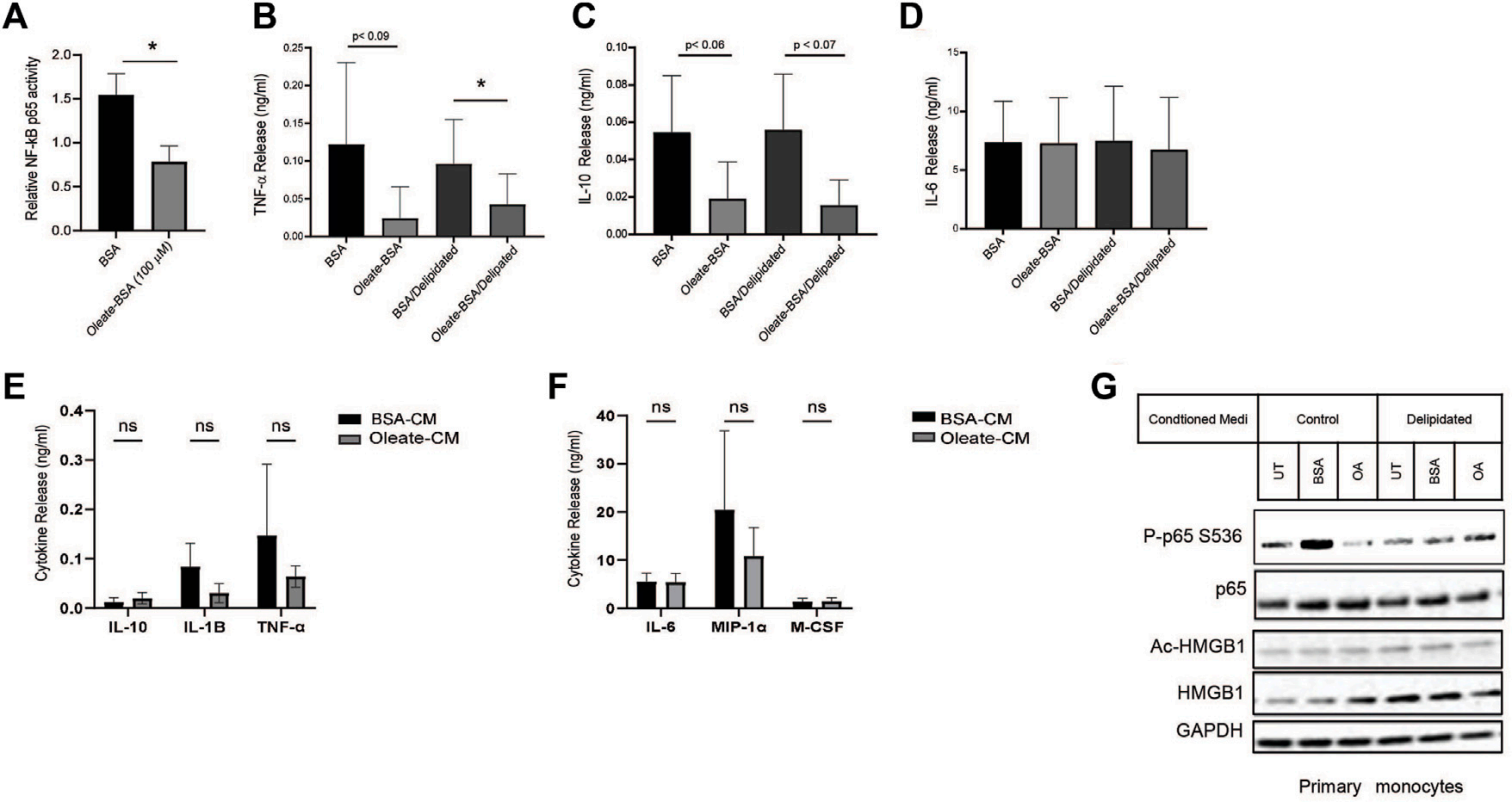
*MUFA suppresses NF-kB-dependent cytokine release from NSCLC cells and monocytes*. **(A)** Relative NF-kB p65 DNA binding in A549 cells delipidated for 16 h and replenished with 100 µM oleate-BSA for 4 h. **(B–D)** Release of cytokines from A549 cells treated with 100 uM oleate-BSA alone, or with delipidation and replenishment with oleate-BSA. **(E)** Release of IL-1-beta, TNF-α, and IL-10 from THP-1 monocytes following 24-h exposure to conditioned from delipidated and oleate-BSA replenished A549 cells. **(F)** Release of IL-6, MIP-1 alpha, and M-CSF from THP-1 monocytes following 24-h exposure to delipidated A549 conditioned media. **(G)** Immunoblot protein analysis on primary monocytes following 24-h exposure to delipidated A549 conditioned media. Error bars represent the standard error of the mean (S.E.M.), an *, indicates a *p*-value < 0.05.

Altering the availability of MUFA has revealed a relationship between fatty acids and protein expression in lung cancer cells. Unsaturated lipids are known to suppress inflammation in cells and tissues, this is in part associated with UFA reversing ER stress (Volmer et al., 2013). The transcription factor NF-kB is a significant driver of many inflammatory genes such as TNF-α, IL-1, and others. To find whether NF-kB was affected by the availability of MUFA, we measured the NF-kB response in lung cancer cells in the presence and absence of MUFA. Following delipidation, replenishment of MUFA significantly decreased the amount of DNA-bound nuclear NF-kB p65 (Figure 6A). We next confirmed that the decrease in nuclear NF-kB p65 was associated with changes in cytokine profiles via ELISA. We observed that MUFA decreased the release of TNF-α, with and without delipidation (Figure 6B). There was also oleate-dependent decrease in secreted IL-10 that failed to reach statistical significance (Figure 6C). Interestingly, there was no apparent effect that oleate had on IL-6 secretion (Figure 6D). The lack of a response in IL-6 may be due to differential regulation, as STAT transcription factors can transcriptionally regulate IL-6. This led us to think that part of the effect MUFA has on lung cancer cells involves regulating inflammatory signaling via HMGB1.

### MUFA suppresses inflammatory signaling in monocytes

Cancer cells do not act in a vacuum and often communicate with the surrounding stromal and immune cells to create an immune microenvironment that supports tumor growth. Using conditioned media from delipidated lung cancer cells we investigated its effect on monocytic cell lines as a model of the interaction between lung cancer cells and innate immune cells in the tumor microenvironment. Conditioned media from MUFA-treated lung cancer cells appeared to suppress secretion of TNF-α, IL-1β, and MIP-1 cytokines from monocytes, but none reached statistical significance (Figures 6E, F). Additionally, conditioned media from MUFA-treated lung cancer cells had little apparent effect on the release of IL-10, M-CSF, and IL-6 from monocytes, which suggest that MUFA does not lead to a complete shift in the cytokine profile (Figures 6E, F). Next, we took patient-derived monocytes to see the impact of cancer cell conditioned media on primary immune cells. Interestingly, primary monocytes only appeared to show concomitant decrease activation of p65 and increased cellular retention of HMGB1 in cells exposed to non-delipidated conditioned media (Figure 6G). This suggests that primary monocyte response is not dependent on prior lipid deprivation. Together these results show that the availability of MUFA to lung cancer cells can suppress monocyte/macrophage behavior in a manner that influences the tumor microenvironment and subsequent immunogenic behavior.

### Decreased HMGB1 protein diminishes conditioned media effect on monocytes

**FIGURE 7:**
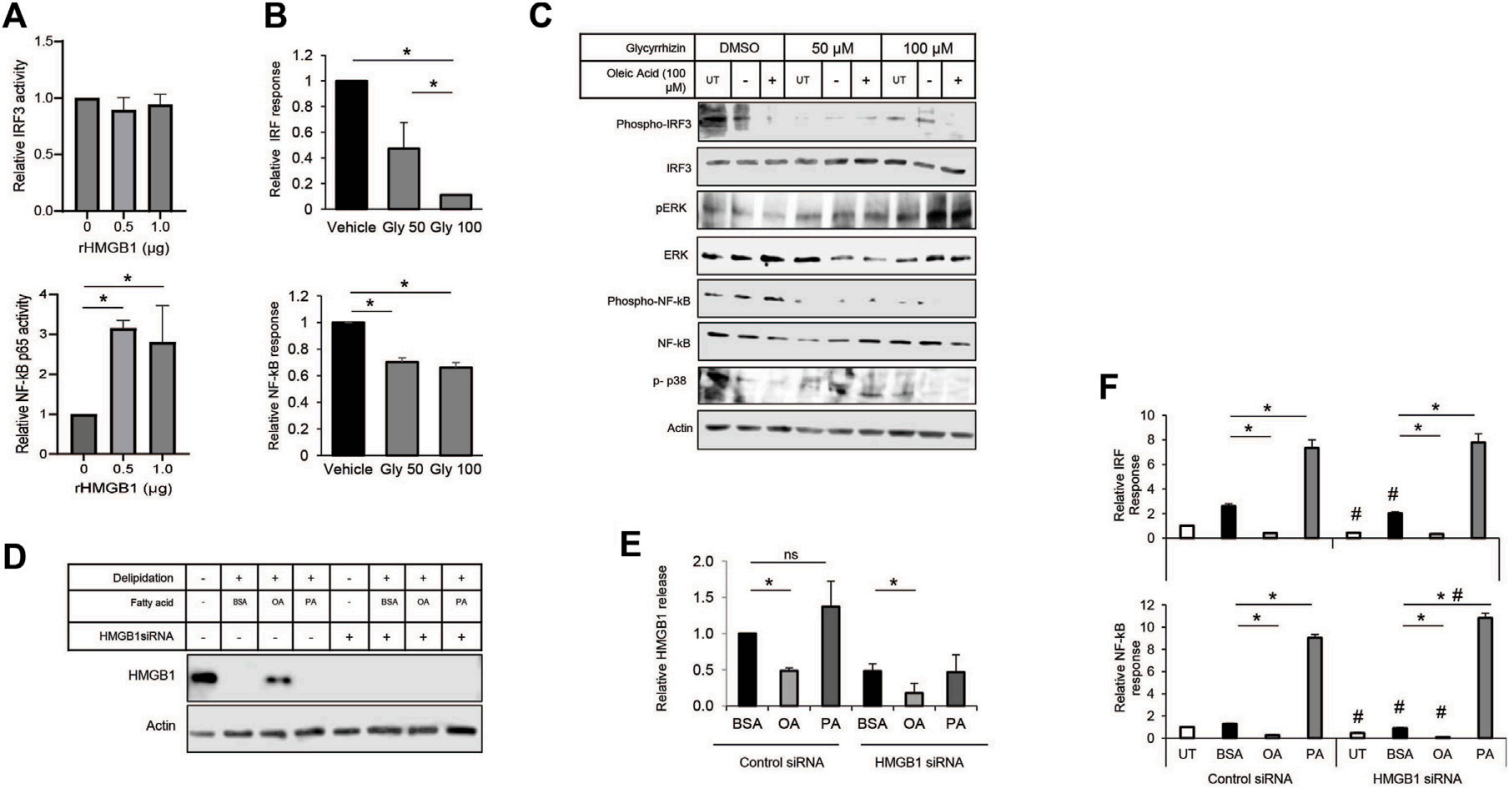
*Modulation of HMGB1 diminishes impact of A549-cond*. *Media on monocytes*. **(A)** THP-1 NF-kB-SEAP reporter cell assay following 24-h incubation with conditioned media from lung cancer cells treated with increasing concentrations of recombinant HMGB1 protein. **(B)** THP-1 IRF3 and NF-kB reporter cell assay following exposure to conditioned media from A549 lung cancer cells treated with glycyrrhizin. **(C)** Immunoblot protein analysis of ERK, NF-kB, p38 signaling pathways in THP-1 cells treated with glycyrrhizin in the presence or absence of MUFA. **(D)** Immunoblot protein analysis of delipidated A549 cells replenished with BSA, oleic acid-BSA, or palmitic acid-BSA; 72 h after transfection with HMGB1-specific siRNA. **(E)** HMGB1-specific ELISA on extracellular media from cells described in Figure 7D. **(F)** THP-1 IRF3-Luc and NF-kB-SEAP reporter assays following 24-h incubation of reporter cells with conditioned media from A549 cells described in Figure 7D. Error bars represent the standard error of the mean (S.E.M.), an *, indicates a *p*-value < 0.05 when comparing the sample to BSA control; a #, indicates a *p*-value < 0.05 when comparing the sample to the control siRNA counterpart.

**FIGURE 8:**
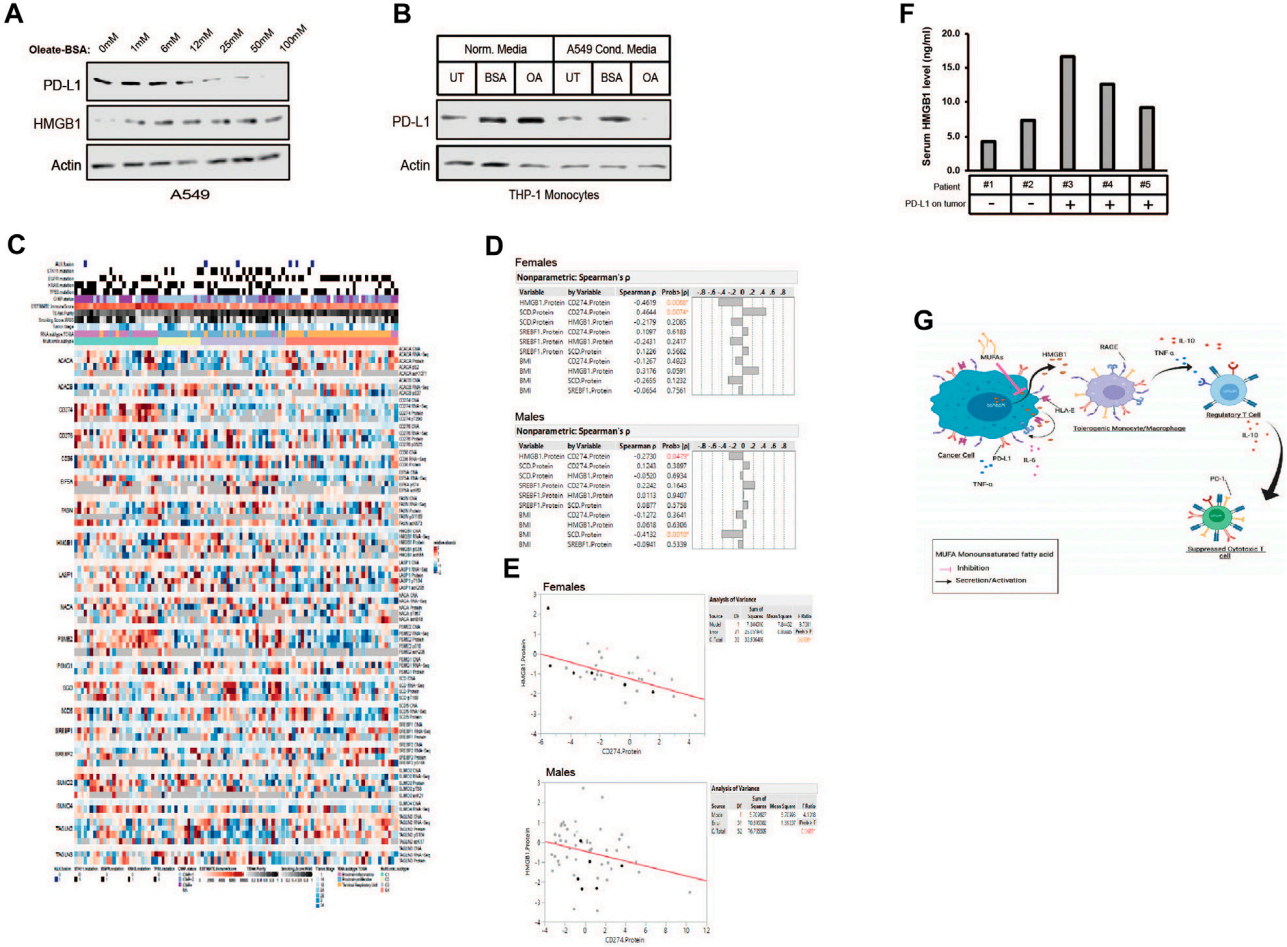
*MUFA increases cell-associated HMGB1 and decreases PD-L1 in NSCLC and monocytes*. **(A)** Immunoblot protein analysis of HMGB1 and PD-L1 expression in A549 cells treated for 4 h with increasing concentrations of oleate-BSA. **(B)** Immunoblot protein analysis of PD-L1 expression in THP-1 monocytes following 24-h incubation in presence or absence of delipidated A549 conditioned media. **(C)** Heatmap of multi-omic data from lung adenocarcinoma published dataset examining expression of HMGB1, SCD1, SREBF1, and PD-L1 (CD274) along with several lipogenic genes and top hits from our proteomic analysis from Figure 2A. **(D)** Spearman’s correlation analysis among the select proteins from **(C)** and body mass index in female and male lung adenocarcinoma patients. **(E)** Pearson correlative analysis between expression of HMGB1 protein and PD-L1 (CD274) in female and male lung adenocarcinoma patients. **(F)** HMGB1-specific ELISA on serum samples from lung adenocarcinoma patients (n = 5), compared to histological detection of tumor-associated PD-L1. Any signal was considered positive for PD-L1 expression using the clinically validated Ventana assay. **(G)** Proposed mechanism of MUFA-mediated suppression of HMGB1 immune modulation in the tumor microenvironment. Error bars represent the standard error of the mean (S.E.M.), an *, indicates a *p*-value < 0.05 when comparing the sample to control.

HMGB1 in the immune response has a variety of interactions with receptors, including the TLRs and RAGE. The multiple binding partners allow HMGB1 to be involved with various pathogen and damage signaling responses. Therefore, we decided to use a monocyte NF-kB/IRF3 reporter cell system to show how each pathway contributes to the MUFA-HMGB1 influence on the immune response in lung cancer. We used genetic and pharmacological methods to inhibit the HMGB1 protein present in conditioned media from MUFA-treated lung cells. To demonstrate the impact of HMGB1 on the activity of our reporter, we treated THP-1 monocytes with recombinant HMGB1 protein. Following incubation there was a significant increase in the NF-kB p65 response in our cells, but trivial difference in IRF3 response (Figure 7A). To measure the impact of endogenous HMGB1 protein on the reporters, we used the natural HMGB1 inhibitor glycyrrhizin to block the function of HMGB1 present in lung cancer cell conditioned media. The addition of glycyrrhizin led to a decrease in both reporters, surprisingly, the effect was only dose-dependent for IRF3 reporter (Figure 7B). Immunoblot analysis and reporter assays confirmed suppression of the IRF3 and NF-kB pathways, but the analysis of additional signaling pathways revealed that glycyrrhizin differentially affected NF-kB, p38, and ERK signaling. MUFA appeared to suppress IRF3, ERK, p38 phosphorylation (Figure 7C). However, the addition glycyrrhizin appeared to suppress ERK at lower concentrations but activated it at higher doses. In fact, in earlier studies, treatment of cells with glycyrrhizin has been shown to activate the autophagy pathway in a ERK-dependent manner. This suggests that the differential impact of glycyrrhizin on IRF3 and NF-kB are likely a convergence of multiple pathways being altered simultaneously by the inhibitor. Therefore, to address the pleiotropic effect of glycyrrhizin, we decided to use siRNA to specifically decrease the levels of endogenous HMGB1 in lung cancer cells. Knockdown with siRNA led to a significant decrease in total HMGB1 protein in lung cancer cells (Figure 7D). Successful knockdown made it difficult to detect MUFA-dependent retention of cell-associated HMGB1. However, quantification of extracellular HMGB1 were not adversely affect to the same extent. Cellular depletion of HMGB1 led to a decrease in HMGB1 released from cells; however, HMGB1 release from HMGB1 knockdown cells remained as sensitive to the addition of free fatty acids as controls (Figure 8E). To measure the effect of HMGB1-depleted lung cancer cells on monocytic cells, we incubated our monocyte reporter cells with HMGB1-depleted lung cancer-conditioned media. MUFA-conditioned media suppressed the stimulation of the IRF3 and NF-kB reporters, while saturated fatty acid, palmitic acid significantly stimulated both reporters (Figure 7F). Strikingly, HMGB1 knockdown significantly suppressed IRF3 and NF-kB activity in all conditions (except saturated fatty acid treatment), but NF-kB activity was further reduced with the addition of oleate acid (Figure 7F lower). This suggests that the effect of oleic acid and HMGB1 knockdown are redundant in IRF3 signaling but not necessarily for NF-kB.

### MUFA increases cellular retention of HMGB1 and decreases PD-L1 expression in lung cancer cells

As we began to view HMGB1 as a MUFA-regulated protein, it became essential to identify its role in the tumor immune landscape. Others have shown that HMGB1 regulates programmed death receptor ligand (PD-L1), a significant target for immune checkpoint therapy (Wang et al., 2019). We wanted to determine whether MUFA could also control the expression of PD-L1 via the regulation of HMGB1. To examine the relationship between MUFA and PD-L1, we exposed lung cancer cells to increasing concentrations of MUFA and measured protein expression. We saw that PD-L1 levels diminished as the concentration of MUFA was increased (Figure 8A). Next, we explored how conditioned media from MUFA-treated cancer cells affected PD-L1 expression in immune cells. We exposed monocytes to conditioned media from MUFA-treated lung cancer cells and measured protein expression the following day. Monocytes exposed directly to MUFA had little noticeable change in PD-L1 expression, but monocytes exposed to MUFA-treated cancer cell-conditioned media had dramatically decreased PD-L1 (Figure 8B). The relative decrease in PD-L1 observed in the monocytes supports the hypothesis that MUFA availability affects the ability of cancer cells to influence the immune response in a paracrine-like manner.

### HMGB1 is negatively correlated with PD-L1 expression in NSCLC patients

Our *in vitro* studies suggest that the availability of MUFA would affect the expression of PD-L1 in patient tumors. To investigate this hypothesis, we analyzed the expression of lipogenic genes, body mass index, and CD274 (PD-L1) levels from published multi-omics lung adenocarcinoma datasets (Figure 8C). Multivariate analysis revealed several significant relationships. Among proteins involved in lipid regulation of HMGB1 we initially focused on desaturase and the relationship with PD-L1 expression. We saw a positive correlation between SCD1 and PD-L1 protein expression in patients; however, significance was only seen amongst the female patients (Figure 8D). Additional analysis led to the identification of a significant negative correlation seen between HMGB1 and PD-L1 protein in both male and female patients (Figures 8D, E). Interestingly, negative correlation between HMGB1 and PD-L1 in tumors was again strongest among female compared to male patients (Figure 8E). Together with our *in vitro* data, we believe this observation was due to the absence of HMGB1 in the cell following its secretion and binding to receptors on nearby cells.

Although the relationship between SCD1 and PD-L1 initially appeared to contradict our first supposition that SCD1 (MUFA) would be negatively related to CD274 (PD-L1) in patients, it may simply indicate a more complex relationship exists. We also observed a negative relationship between HMGB1 and SCD1, in which additional analysis across cancer sub-types further revealed more dramatic statistical differences between HMGB1 and SCD1 within the “proximal inflammatory” and “terminal respiratory unit” subtypes (not shown) (Collisson et al., 2014). Lastly, as there exists some debate around the role of obesity or elevated BMI and patient prognosis and response to treatment, we also wanted to analyze the relationship between BMI and proteins of interest. Interestingly, we saw a negative relationship between SCD1 and BMI, especially in male patients (Figure 8D). This suggests the possibility that individuals with elevated BMI also have lower SCD1 protein, thus less MUFA available to prevent HMGB1 release, which could drive immune suppression.

To confirm the observed correlation between HMGB1 and PD-L1 in the above dataset, we collected patient serum and tissues to analyze the level of secreted HMGB1 in patients with PD-L1-positive tumors. From the 14 patients enrolled, we obtained serum, tissue, and pathology reports for only five. We measured the amount of HMGB1 in the patient’s serum using a human HMGB1 ELISA. Patients with tumors that were negative for PD-L1 based on immunohistochemical analysis tended to have less HMGB1 in their blood when compared to patients with PD-L1-positive tumors (Figure 8F). Acquisition and analysis of additional patient tissue and blood from multiple sites are in progress to increase the statistical power of our observations. Withstanding the small sample size, the data we have generated suggests that the relationship between HMGB1, SCD, and PD-L1 may help clinicians stratify patients into groups based on the potential to predict the immunological sensitivity of tumors to current and future immunotherapies.

## Discussion

In this study, we have uncovered a complex interplay between lipid metabolism, the release of HMGB1, and immune modulation within the context of NSCLC. Our findings highlight the critical role of SCD1 and MUFA in regulating essential elements of the tumor microenvironment, shedding light on potential therapeutic strategies and avenues for further investigation.

Our results showed that a significant decrease in the expression of SCD1 exists in NSCLC tumors compared to normal tissues. This finding underscores the importance of lipid metabolism in the context of lung cancer, a concept supported by earlier work from our laboratory and others. The intricacies of *de novo* synthesis and uptake of MUFA are likely to play pivotal roles in developing and supporting lung tumors. Although the influence of SCD1 expression on patient survival was primarily clear in specific population quartiles, indicating an outlier effect, the observed disparity between SCD1 expression in normal and malignant tissues cannot be overlooked. These results are evidence of the impact of SCD1 and lipid metabolism on tumor progression.

Our results also showed that lipid metabolism plays a significant role in HMGB1 and the immune response to lung cancer. Inhibiting HMGB1 decreased the expression of distinct immune response-related genes, particularly those regulated by NF-kB signaling, affirming that HMGB1 is a critical mediator in the communication between lung cancer cells and the immune system.

Notably, our study unveiled a connection between MUFA availability and the regulation of PD-L1 expression in lung cancer cells, a pivotal target for immunotherapy. PD-L1 expression decreased as the concentration of MUFA increased, showing a potential avenue for modulating the immune response. Furthermore, the combination of our *in vitro* and patient data suggested a multifaceted relationship between cancer and immune cells in the tumor microenvironment that hinge on interactions among MUFA, HMGB1 and PD-L1, potentially influencing the immunological sensitivity of tumors to current and future immunotherapies (Figure 8G).

In conclusion, these findings open doors to further investigations and the development of innovative therapeutic strategies that target key elements of this intricate interplay. Understanding the regulation of lipid metabolism and immune responses in lung cancer is crucial for developing more effective treatments and personalized approaches for patients. As we continue to uncover the complexities of these interactions, we hope to contribute to the ever-advancing field of cancer research and the eventual improvement of patient outcomes.

## Materials and methods

### Cell culture

A549, HCC827, H1299, H23 non-small cell lung carcinoma cells were purchased from ATCC. A549 and H1299 cells were cultured and maintained in low glucose DMEM (Corning cat #10-014-CV) supplemented with 5% FBS, 50 U/mL penicillin, 50 μg ml/mL streptomycin. HCC827 and H23 cells were cultured and maintained in low glucose DMEM supplemented with 10% FBS, 50 U/mL penicillin, 50 μg mL/mL streptomycin. THP1-Dual cells (InvivoGen) were cultured and maintained in RPMI 1640 (Corning) supplemented with 10% FBS, 50 U/mL penicillin, 50 μg mL/mL streptomycin, 100 μg/mL normocin, and 2 mM L-glutamine. 100 μg/mL of zeocin and 10 μg/mL of blasticidin were added to growth medium every other passage to retain the dual reporters.

### Materials

Antibodies used in this study were the following: For immunoblotting, mouse monoclonal β-catenin (1:10,000; MilliporeSigma, SAB1305546), rabbit polyclonal HMGB1 (1:2,500; Novus, NB100-2322), mouse monoclonal PD-L1 (1:3,000; Sino Biologicals, 10084-MM33-50), rabbit monoclonal phospho-IRF3 (1:1,000, Cell Signaling 37829), rabbit polyclonal IRF3 (1:10,000, ProteinTech 11312-AP), rabbit monoclonal NF-kappa B p65 (1:1,000, Cell Signaling 8242), rabbit monoclonal phospho-NF-kappa B p65 (1:1,000, Cell Signaling, 3033). For immunoprecipitation, rabbit polyclonal HMGB1 (3ug; Abcam, ab18256), mouse monoclonal Acetyl-lysine (1:200; Cayman, 10010567). Fatty acids, sodium oleate (cat #: 07501), sodium palmitate (cat #: p9767), and sodium arachidonate (cat #: SML1395) were all purchased from MilliporeSigma, Goat anti-Mouse and anti-Rabbit secondary antibodies were purchased from LiCOR (cat #: 926-80010 and 926-80011). To make 10 mM stock solutions of each fatty acid purchased from MilliporeSigma (cat # O7501-1G, P9767-5G, SML1395-25 MG), we conjugated fatty acids to bovine serum albumin by taking the sodium salts of individual fatty acids adding it to a 0.9% sodium chloride solution. Resuspended fatty acid was combined with 24% fatty acid-free BSA and mixed on a stir plate until fully resuspended while under inert gas (some gentle heating was necessary for palmitate-BSA resuspension). The 10 mM fatty acid-BSA mixtures were then sterilized through a 0.2 mm filter. The stock solutions were stored in 300 µL aliquots passed under nitrogen gas to prevent oxidation. 250 mM stocks of glycyrrhizin, an HMGB1 inhibitor was made with DMSO from was purchased glycyrrhizic acid purchased from Cayman Chemicals (cat #: 11847).

### Lipid deprivation

Lung cancer cells were plated in 60 mm (250,000 cells) or 100 mm (700,000 cells) tissue culture dishes in complete DMEM as described above for specific experimental conditions overnight. The following day, all culture media was removed, and cells were rinsed one time with sterile phosphate buffered saline. Cells were replenished with delipidation media (DMEM supplemented with 100 units/mL penicillin, and 100 μg/mL streptomycin with 5% delipidated fetal bovine serum (DFBS, GeminiBio 900-123), and 1 µM SCD-1 inhibitor (A939572, Cayman Chemicals)). Cells were incubated in delipidation media overnight and assayed for response to lipids the following day. The lipid replenishment was carried by adding the desired concentration of specified fatty acids (oleate, palmitate, or arachidonate) for 4 h. For single treatments, 100 µM oleate-BSA, palmitate-BSA, or arachidonate-BSA were used for experiments described in this study.

### Cell harvest and lysis

Cells were harvested by scraping the cells and media into conical tubes and placing them on ice. Following harvest, the cell suspensions were spun down at room temperature for 5 min at 800 × g. The supernatant was aspirated, and the cells were washed twice with PBS. Cells were then lysed with 100 µL of seize2 lysis buffer (25 mM Tris-HCl pH 7.2, 0.15 M NaCl, 1% NP-40, and 1x cOmplete EDTA-free protease inhibitor cocktail (Roche cat no. 4693132001). The lysate was passed through a 22 G needle ten times while on ice followed by rotation at 4°C for 15 min. The lysate was pelleted at 21,000 × g for 10 min and the supernatant was collected. Protein concentrations were found using the BCA Protein Assay kit (Thermo Fisher Scientific) and read at 562 nm. Sample lysate was mixed with loading buffer and then boiled for 5 min and loaded onto 10% SDS-PAGE gel at 10 or 20 µg total protein per well.

### Immunoblot analysis

Following overnight treatment with fatty acid modulation, cells were harvested and lysed as previously described. A total of 10 µg of protein was then separated by 10% SDS-PAGE. After gel electrophoresis was completed, the proteins were transferred to a 0.2-micron polyvinylidene difluoride (PVDF) membrane. All immunoblot equipment and reagents used were from Bio-Rad. The membrane was blocked with a 5% non-fat milk/PBST solution for 30 min. The membrane was incubated with primary antibodies at 4°C overnight while rocking. The following day the membrane was washed three times with PBST at 5 min per wash. Then 5% non-fat milk/PBST containing HRP-conjugated secondary was added to the membrane. β-actin was used as the loading control for all immunoblots (primary antibodies: Sigma, 1:2,500 dilution; secondary antibody. Protein bands were detected by using 1 mL of SuperSignal ECL from Pierce and visualizing band intensity using Li-Cor C-Digit imaging system.

### Cell viability

For the CCK-8 assay, A549 cells were seeded at a density of 1,000 cells/well in 96-well plates and treated with or without 1 µM SCD1 inhibitor for 24 h. Subsequently, 10 μL of CCK-8 solution (Abcam, cat no. ab228554) was added to each well, and the plates were incubated at 37 °C for 2 h. Finally, the absorbance was measured at 450 nm.

### Plasmid transfection

A549 cells were seeded in 60 mm plates then transfected with 2 µg of N-terminal tagged GFP-HMGB1 DNA plasmid DNA (Sino biological, cat no. HG10326-ANG). In brief, 250,000 cells were plated in 60 mm dishes the night prior to transfection and placed at 37°C. Transfection was done using X-tremeGENE HP DNA transfection reagent (Roche, cat no. 6366244001). Each dish was transfected with 2 µg of plasmid DNA per manufacturer’s instructions. The GFP signal was confirmed using a CellDrop Automated Fluorescent Cell Counter (DeNovix) to confirm protein expression prior to imaging on the EVOS M5000 fluorescent imaging system (ThermoFisher, cat no. AMF5000). Cells were incubated 24 h before harvest to analyze protein and mRNA expression.

### Fluorescent microscopy

Following treatment of HMGB1-GFP transfected A549 cells with oleate-BSA cells were stained with Nile Red. In brief, stock solution of Nile Red (1 mg/mL in DMSO) was diluted to 1 μg/mL. In a 24-well plate while using only 12 wells, cells were washed twice with sterile PBS and fixed with 3.7% formaldehyde for 15 min at room temperature. Formaldehyde was washed away with two PBS washes before staining with Nile Red working solution and incubating for 30 min. The cells were washed with PBS once more and then imaged on EVOS M500 imaging system using ×20 magnification.

### RNA interference

150,000 cells were plated in 60 mm dishes overnight at 37°C. Following day transfection mixes were made for each experimental permutation. SiGENOME SMARTpool siRNA for HMGB1 (M-018981-01-0005), SIRT1 (M-003540-01-0005), and control non-targeting siRNA pool #1 (D-001206-13-05) were transfected 100 nM per dish. Lipofectamine RNAiMax (Invitrogen Cat: 13778030) was mixed with appropriate volume of OptiMEM (Gibco) in one tube, while in a separate tube the appropriate amount of each siRNA was added to appropriate volume of OptiMEM (Gibco, cat no. 31985070) and mixed. Individual tubes were incubated at room temperature for 5 min. The two solutions were combined at equal volume, mixed, and incubated at room temperature for 20 min. Plates containing cells were washed, and replenished with 1.6 mL of OptiMEM, and placed back at 37°C until transfection complexes were ready. Then, 400 µL of transfection mix was added to each dish, and incubated at 37°C for 4 h, at which cells would be washed once with complete media and 4 mL of complete media would be added to cells. Cells then incubated at 37°C for 48 h.

### Reverse transcriptase quantitative polymerase chain reaction

24-h post transfection with siRNAs as described above. Total RNA was obtained from cells by Qiazol extraction and RNeasy purification (Qiagen, cat no. 74104). HMGB1 and SCD1 mRNA levels were determined using RT-qPCR using a Rotor-gene Q PCR machine. Reverse transcription step was conducted using the miScript II RT kit (Qiagen, cat #: 218160) according to the manufacturer’s protocol. The data were analyzed using the 2^−ΔΔCq^ method and the Actin mRNA was used as an endogenous control as previously described (Livak and Schmittgen, 2001). The following primers used for quantitative PCR are as follows: HMGB1 Forward: 5′-AAA GGA TAT TGC TGC ATA TCG AGC TAA AGG A-3′, Reverse: 5′-CCT CAT CCT CTT CAT CTT CCT CAT CTT CC-3′; Actin Forward 5′-ATC CAC GAA ACT ACC TTC AAC TC-3′, Reverse: 5′-GAG GAG CAA TGA TCT TGA TCT TC-3′; SCD forward: 5′-GTT CCA GAG GAG GTA CTA CAA ACC TGG-3′, Reverse: 5′-GTA GTT GTG GAA GCC CTC ACC CA-3′.

### Lucia luciferase and SEAP assays

QUANTI-Luc luciferase reagent (InvivoGen, rep-qlc4lg5) was used to measure IRF activity through Luciferase luminescence following the manufacturer’s protocol. To measure IRF, 20 μL of cell culture supernatant was transferred to 96-well opaque plate and luminescence was read using a Biotek Synergy spectrophotometer after addition of 50 μL of luciferase reagent. NF-kB activity was measured by combining 20 μL of supernatant with180 μL QUANTI-Blue SEAP detection reagent (InvivoGen, cat no. rep-qbs) in 96-well assay plate. The samples were then incubated at 37°C for 1 h and absorbance was measured at 650 nm.

### Statistical analysis

All experiments were performed in triplicate (n = 3), unless otherwise indicated. An unpaired two-tailed Student’s t-test with two degrees of freedom was used to compare means of the three replicate experiments between treatments using either GraphPad Prism or MS Excel. Where appropriate, the Bonferroni correction was applied to t-tests. *p* < 0.05 was considered to indicate a statistically significant difference. Association between OS and key proteins was determined by univariable Cox proportional Hazard models. Associations between key proteins of interest were determined by multivariate pairwise analysis using Spearman ranked correlations for each pair of set of variables (*p* < 0.05 implies a statistically significant marginal association at the 0.05 alpha level). Multivariate analysis was done using proportion of pairwise correlations in JMP software by SAS. Far publicly available patient data, the method for differential analysis is one-way ANOVA, using disease state (Tumor or Normal) as variable for calculating differential expression. The expression data are first log 2 (TPM^+1^) transformed for differential analysis and the log 2FC is defined as median (Tumor) - median (Normal). Genes with higher |log 2FC| values and lower q values than pre-set thresholds are considered differentially expressed genes.

## Data Availability

The original contributions presented in the study are included in the article/Supplementary Materials, further inquiries can be directed to the corresponding author.
