# Correction: Oleate alters the immune response in non-small cell lung adenocarcinoma through regulation of HMGB1 release

**DOI:** 10.3389/fcell.2026.1852559

**Published:** 2026-05-01

**Authors:** Breanna Cole-Skinner, Nicole M. Andre, Zachary Blankenheim, Kate M. Root, Kisa Jafri, Glenn E. Simmons

**Affiliations:** 1 Department of Molecular Microbiology and Immunology, University of Missouri, Columbia, United States; 2 Department of Biomedical Sciences, College of Veterinary Medicine, Cornell University, Ithaca, United States; 3 Department of Biomedical Sciences, School of Medicine, University of Minnesota, Duluth, United States

**Keywords:** HMGB1 (high mobility group box 1), lung cancer, tumor microenvironment, fatty acid, immunotherapy

There was a mistake in Figure 5A as published. Experiments that were carried out on H1299 and H23 lung cancer cell lines were not carried out as described for A549 and HCC827. The corrected Figure 5A appears below, with the removal of those two cell lines.

**FIGURE 5:**
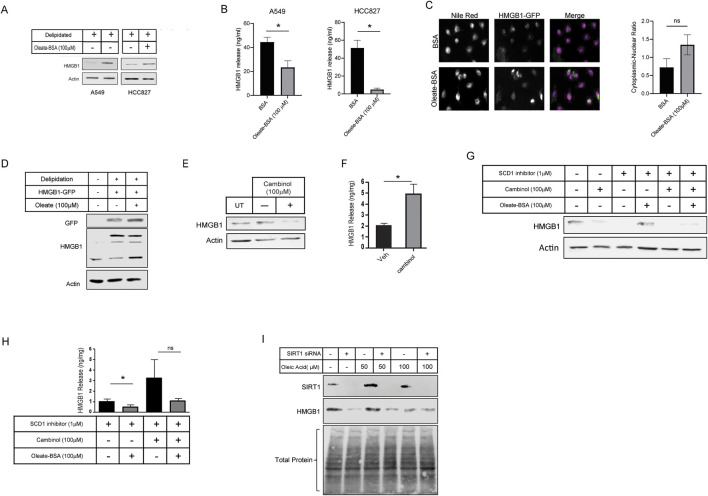
MUFA increases retention of HMGB1 in lung cancer cells in a SIRT-dependent manner. **(A)** Immunoblot protein analysis of KRAS G12S mutant (A549) and EGFR deletion mutant (HCC827) lung cancer cells lines treated with delipidation media (delipidated serum and 1 µM SCD1 inhibitor) and 4-h replenishment with oleate-BSA. **(B)** HMGB1-specific ELISA on extracellular media from A549 and HCC827 lung cancer cells following 16-h delipidation and 4-h oleate-BSA replenishment. **(C)** Fluorescence microscopy of HMGB1-GFP transfected A549 cells following 16-h delipidation and 4-h oleate-BSA replenishment and neutral lipid staining with Nile Red, along with quantitation of localization of HMGB1-GFP sig nals. **(D)** Immunoblot protein analysis of HMGB1-GFP transfected A549 cells from **(C)**. **(E)** Immunoblot protein analysis of A549 lung cancer cells treated SIRT1 inhibitor cambinol for 24-h. **(F)** HMGB1-specific ELISA on extracellular media from cells treated in **(E)**. **(G)** Immunoblot protein analysis of A549 cells treated with combination of cambinol and SCD1 inhibitor in the presence and oleateBSA. **(H)** HMGB1-specific ELISA on extracellular media obtained from cells treated with SIRT1 and SCD1 inhibitors in **(G)**. **(I)** Immunoblot protein analysis of delipidated A549 cells replenished with oleic acid-BSA, 72 h after transfection with SIRT1-specific siRNA. Error bars represent the standard error of the mean (S.E.M.), an *, indicates a p-value < 0.05.

There was a mistake in the caption of Figure 5A as published. The caption for Figure 5A originally stated “MUFA increases retention of HMGB1 in lung cancer cells in a SIRT-dependent manner. (A) Immunoblot protein analysis of multiple lung cancer cells lines treated with delipidation media (delipidated serum and 1 µM SCD1 inhibitor) and 4-h replenishment with oleate-BSA”. The corrected caption of Figure 5A appears below.

The original article has been updated.

